# Value of collaborative investigation by hospital infection control, public health services and a national reference laboratory during an increase in puerperal sepsis

**DOI:** 10.1186/s13756-025-01564-z

**Published:** 2025-05-28

**Authors:** Irene V. Hoogendijk, Diane de Zwart - Slats, Stefan A. Boers, Boas C.L. van der Putten, Nina M. van Sorge, Bibi D.H. Molenaar, Myrthe M.A. Toorop, Marieke B. Veenhof, Karin Ellen Veldkamp, Adriënne S. van der Schoor, Joffrey van Prehn

**Affiliations:** 1 Department of Medical Microbiology and Infection Prevention, Centre for Infectious Diseases (LU-CID), Leiden University, Leiden University Medical Centre, Leiden, The Netherlands; 2 Department of infectious disease, Public Health Service region Hollands Midden, Leiden, The Netherlands; 3 Department of Medical Microbiology and Infection Prevention, Netherlands Reference Laboratory for Bacterial Meningitis, Amsterdam University Medical Centre, Amsterdam, The Netherlands; 4 Department of Occupational Medicine, Leiden University Medical Centre, Leiden, The Netherlands; 5 Department of Obstetrics, Leiden University Medical Centre, Leiden, The Netherlands

**Keywords:** *S. pyogenes*, Group A *Streptococcus*, Infection, Puerperal sepsis, Molecular typing, Whole-Genome sequencing, Core genome multilocus sequence typing (cgMLST), Outbreak investigation, Public health, National reference laboratory

## Abstract

**Background:**

In a Dutch tertiary care hospital, two cases of puerperal sepsis were diagnosed within 16 days in June-July 2022. The subsequent outbreak investigation emphasizes the value of collaboration between hospital infection control, regional public health services (PHS) and a national reference laboratory. The aim was to identify possible causes of this increase to prevent further cases of puerperal sepsis.

**Methods:**

Hospital infection control identified a group of puerperal sepsis cases clustered within the last year in the hospital, a cluster caused by *S. pyogenes emm*12.0. The hospital and PHS performed contact tracing of cases and HCW involved, investigating epidemiological links, and screening of HCW. The Netherlands Reference Laboratory for Bacterial Meningitis (NRLBM) identified additional regional cases. Subsequently, whole genome sequencing (WGS) analysis was performed on clinical, HCW and regional *S. pyogenes* isolates.

**Results:**

Four maternity ward patients were diagnosed with puerperal sepsis caused by *S. pyogenes emm*12.0 between April and November 2022. Although no additional epidemiological links were identified, all four cases resided within a 6.6 km radius. WGS analysis showed that the four cases were part of an 11-case cluster. Screening of HCW (*n* = 197) identified two individuals carrying clonally related *S. pyogenes* isolates.

**Conclusions:**

Collaboration between hospital, PHS, and NRLBM resulted in an overview of possible epidemiological links. Centralized collection of iGAS case information and strain typing are critical to place hospital clusters in the context of local epidemiology. An increase in healthcare-associated infections may not necessarily imply in-hospital transmission.

**Supplementary Information:**

The online version contains supplementary material available at 10.1186/s13756-025-01564-z.

## Background

*Streptococcus pyogenes*, or group A *Streptococcus* (GAS), is an aerobic gram-positive coccus that is very contagious and readily colonizes mucous membranes in the respiratory, gastrointestinal, and genital tracts, as well as the skin. It is associated with skin infections and pharyngitis, but can cause more invasive infections including puerperal sepsis, necrotizing fasciitis, and toxic shock syndrome (from here referred to as iGAS infection). Transmission of *S. pyogenes* occurs through direct person-to-person contact and exposure to body fluids, mainly originating from nose, throat, or wounds. The source of infection in puerperal sepsis can be a pre-existing colonization of *S. pyogenes* carriage, or colonization after transmission via a close contact or healthcare worker (HCW) [1, 2]. Pregnant and postpartum women have a two-fold and 20-fold increased risk of an invasive GAS infection, respectively [3, 4].

In 2022, an increase in iGAS infections, compared to the pre-COVID-19 era, was observed in the Netherlands by De Gier et al. [5]. This increase was first observed in children between ages 0–5 years, followed by an increase in iGAS infections in adults. This increase was also observed throughout Europe [6–10]. In April 2022, *emm*12.0 was the dominant *emm* type [5]. Later that year, *emm*12.0 proportion decreased to around 5% in December, while *emm*1 predominantly M1UK became dominant accounting for over 60% of all typed isolates [11].

In the Netherlands, iGAS infection has been notifiable by law since 2008: upon diagnosis the medical microbiology laboratory and treating physician are obliged to notify the regional public health service (PHS) within one working day. More broadly, *S. pyogenes* isolates from all different invasive GAS manifestations are submitted to the NRLBM (Netherlands Reference Laboratory for Bacterial Meningitis, located at Amsterdam University Medical Center) for *emm* typing since 2019. To support outbreak investigations, whole genome sequencing (WGS) followed by core genome multilocus sequence typing (cgMLST) and whole genome single nucleotide polymorphism (wgSNP) analysis can be performed for improved high-resolution typing.

In July 2022 an investigation was initiated after encountering two cases of puerperal sepsis with positive vaginal/cervical GAS cultures within 16 days at the maternity ward of the Leiden University Medical Center (LUMC), a tertiary-care hospital. The investigation grew to include 5 cases occurring between December 2021 and October 2022, four of which were due to *emm*12.0 isolates. Here we describe the infection control measures and findings of the outbreak investigation of hospital infection control unit, PHS and national reference laboratory.

## Methods

**Fig. 1:**
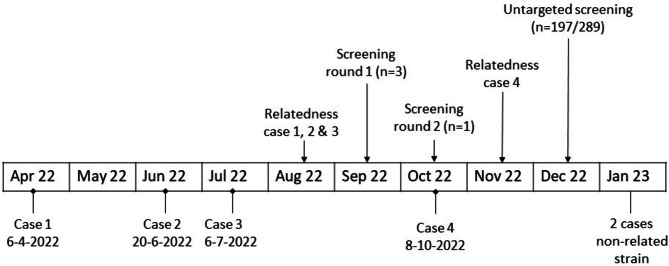
Timeline of key events in outbreak investigation of puerperal sepsis cluster. Screening; screening of health care workers (HCW) for nasal and pharyngeal GAS carriage, 2 rounds of targeted screening of HCW with contact to cases, and untargeted screening of all HCW. Relatedness; at these time points the genetic relatedness of cases was established using core genome multilocus sequence typing (cgMLST) of *S. pyogenes* isolates

In the outbreak investigation cases were defined as women with a clinical diagnosis of puerperal sepsis after having given birth at the maternity ward of the LUMC from July 2021 onwards (the year preceding the two cases that triggered the investigation), caused by *S. pyogenes emm*12.0. A timeline of puerperal sepsis cases and key events of the outbreak investigation is shown in Fig. 1.

### Setting

The maternity ward at LUMC has 9 birthing rooms and hosts an average of 2851 deliveries per year, (data 2021–2022). In 2021 one case of puerperal sepsis was notified to the PHS, before 2021 this ranged from 2 to 3 cases per year (baseline incidence). This ward includes birthing rooms for both in-hospital and outpatient deliveries, the latter supervised by primary care obstetricians. Caesarean sections are performed on the general operating theatre complex of LUMC. During delivery protective gloves are worn.

### Hospital investigations

Immediately after the notification of two patients with puerperal sepsis in July 2022, an outbreak management team (OMT) was initiated by the infection control unit to conduct an outbreak investigation on the maternity ward, obstetrics outpatient clinic, and fertility clinic. The OMT consisted of a clinical microbiologist, infection control expert, a gynecologist, a head nurse, and a head midwife. Potential epidemiological links, such as common hospital rooms, medical procedures and HCW, and routes of transmission were discussed. Proposed potential transmission routes included *S. pyogenes* carriage among HCW and contaminated medical devices. Interrogation about the use, cleaning, disinfection and sterilization, and storage of medical devices took place (e.g., birthing chair, echo probes etc.). Additionally, overlapping contacts with HCW and cases within the hospital and outpatient clinic were identified using the electronic health record.

Later, typing by the NRLBM showed that the two cases were caused by *S. pyogenes emm*12.0. In retrospect two more puerperal sepsis cases were identified at the maternity ward in the preceding year. One in December 2021 with a different *emm* *type* (12.68) and another in April 2022 with an identical *emm*12.0 type. In-house cgMLST typing of GAS isolates of the three *emm*12.0 cases was requested.

### Screening of healthcare workers

After confirmation with cgMLST that the three *emm*12.0 cases were clonally related (sequence type 36, maximum 2 alleles difference) we initiated screening for nasal and pharyngeal GAS carriage (screening round 1). Contact investigation determined that none of the HCW had been in contact with all 3 cases prior to their illness onset dates and therefore HCW that had been in contact with two of the three cases in the period up to their illness were screened for carriage in September 2022. Upon notification of a fourth *emm*12.0 case in October 2022, contact investigation identified one more HCW that had contact with 2 out of the 4 cases and was also screened in October 2022 (screening round 2). Contact was defined as any contact traceable via electronic health record, including in- or outpatient check-ups. Because no HCW with GAS carriage were identified in targeted screening rounds, the screening was broadened and all HCW employed at the maternity ward, obstetrics outpatient clinic, and fertility clinic were invited for screening, which was executed in December 2022 [12]. For culture protocol, see Additional file 1.

### Public health investigations

Upon notification of an individual puerperal sepsis case, the PHS conducts contact tracing, prophylactic treatment and monitoring in the social network around each case. Also, primary care obstetricians and maternity care services involved are informed and asked to record HCW contacts in case of further outbreak investigation. In this particular outbreak investigation, contact tracing between the four cases and the involved HCW was performed. Furthermore, the outbreak investigation included mapping the household, place of residence and work, social network of the cases and their children to find possible epidemiological links between cases, as well as inquiring about related cases with a possible GAS infection that could be a source of infection.

### Molecular typing of isolates

To investigate the possibility of a regional cluster, in collaboration with the NRLBM, all additional *emm*12.0 cases that were presented in 2022 to the NRLBM by the nearest hospitals were identified, resulting in a range of 30 km radius around the hospital. To confirm genetic relatedness of outbreak and regional *emm*12.0 isolates, cgMLST was performed at the diagnostic medical microbiology laboratory of the LUMC [13]. The WGS data was generated using the Illumina MiniSeq platform with 2 × 150 bp chemistry (Illumina, San Diego, CA, USA). The minimum spanning tree (MST) was generated with Ridom SeqSphere + software. Based on cgMLST, strains were part of the cluster in case of < 5 target gene differences out of 1095 target loci [13]. Quality control included contig number < 1000 and contig N50 > 15,000. Percentage of included cgMLST target loci was > 98% for all isolates. Additionally, WGS single nucleotide polymorphism (wgSNP) analysis was performed at the NRLBM using Juno-assembly v2.2.1 and Juno-89 SNP v0.1 [14]. Strains analyzed with wgSNP were deemed part of the cluster in case of SNP distance = < 28.

## Results

### Study population

The four cases (age range 25–39 years) with puerperal sepsis caused by *S. pyogenes* type *emm*12.0 occurred between April 2022 and October 2022. Time between delivery and disease onset ranged from one to four days in three cases and was 15 days in one case. The latter case had been discharged after having given birth and readmitted due to her illness. None of the patients had symptoms indicating any other GAS related infection prior to developing puerperal sepsis. In one case, *S. pyogenes* was detected in a urine sample a week before giving birth.

### Screening of health care workers

Initial targeted screening included three HCW, all gynecologists whether or not in training, whom GAS carriage was not detected. Later, untargeted screening of HCW of maternity ward, obstetrics outpatient clinic, and fertility clinic (*n* = 197 of 289 invited HCW screened, 68% response rate) resulted in eight GAS carriers, a positivity rate of 4.1%. All GAS carriers, independent of GAS-typing results, were treated with azithromycin via occupational health services. Molecular typing results were not disclosed to the individual HCW. No follow up of the treated GAS carriers was conducted. Two of the eight HCW carried the same strain of *S. pyogenes* type *emm*12.0 as the patients. Of those two HCW, one was an assistant on the ward and was likely to have had contact with the cases at some point. The other one was a HCW that was involved before delivery and during readmission of one case.

### Public health investigations

There was no overlap in primary care settings and HCW involved during pregnancy or after childbirth. Place of residence between the four *emm*12.0 cases ranged from 2 to 20 km from each other and was 5–15 km from the hospital. One patient stayed with a relative during the source period post-partum, before developing puerperal sepsis. When linking this stay to place of residence of the other cases, the case region decreases to a 6.6 km radius. One case mentioned a possible case of scarlet fever at the school of her children after which one of her children experienced an upper respiratory tract infection. Another case mentioned contact with a child with a skin infection for which antibiotics were prescribed. But no additional laboratory diagnostics had been performed. No links were discovered upon further investigation of community, work, schools, or social network. Information about the residence of one of the two HCW carriers was disclosed and this HCW resided within the same region as the patients (< 7 km radius).

### Molecular typing

**Fig. 2:**
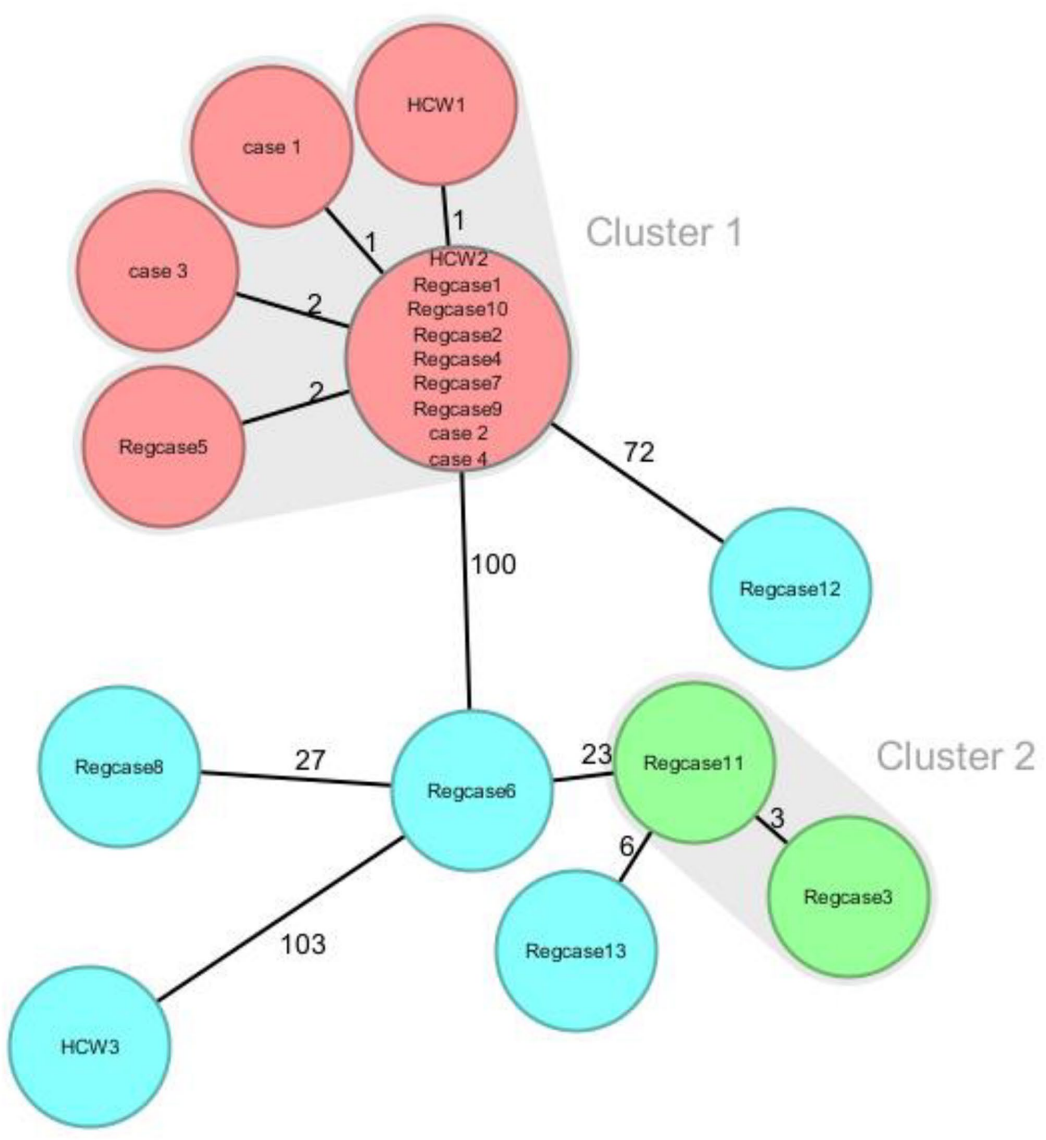
Minimum spanning tree (MST) based on core genome multi locus sequence typing (cgMLST) of *S. pyogenes* isolates from puerperal sepsis cases (case 1–4), three healthcare workers (HCW1-3) and 13 additional regional cases (Regcase1-13). The numbers at the connecting lines indicate the number of target gene differences between isolates. Cluster threshold was set at < 5 target gene differences. Clusters are identified by color: red = regional *S. pyogenes* cluster, green = non-related *S. pyogenes* cluster, and blue = other regional iGAS cases

In 2022, a total number of 1110 *S. pyogenes* strains were presented in the Netherlands to the NRLBM for *emm* typing as part of Dutch surveillance, of which 159 (14.3%) were *emm*12. To investigate the possibility of a regional cluster, all other GAS *emm*12.0 cases in 2022 within a 30 km radius of the hospital were identified (*n* = 13). CgMLST identified that the four puerperal sepsis cases belonged to a cluster of 11 cases **(**Fig. 2**)**. WgSNP analysis yielded similar results and demonstrated the same cluster (Additional file Figure A1). The cluster thus consisted of the four cases and seven regional cases. In this analysis, another cluster of two cases was identified, but this was not related to our cases or cluster. Of the eight GAS carriers identified through HCW screening, two were clonally related to the *emm*12.0 cluster. Altogether, this resulted in a regional cluster of 13 GAS *emm*12.0 cases and carriers.

## Discussion

In total, the outbreak investigation brought to light a cluster of four women diagnosed with puerperal sepsis caused by a clonally related type of *S. pyogenes* type *emm*12.0, after having given birth at the maternity ward of the LUMC between April 2022 and October 2022, as well as two HCW carriers of the same strain who were found during screening in December 2022. All of these cases were part of a regional cluster of thirteen *emm*12.0 cases and carriers, diagnosed in 2022. Despite the extensive efforts to trace a source for the outbreak, no definite nosocomial transmission of *S. pyogenes* was identified. All four HCW initially included tested negative for GAS upon screening. One of the two HCWs colonized with *emm*12.0 found by department-wide screening had direct contact with only one case. Colonization of HCW with the outbreak strain at the hospital could suggest nosocomial transmission. However, other possible sources for the cases may have been community derived [15], as may be seen in over 40% of infections [16], such as skin infections or an asymptomatic colonized carrier. *S. pyogenes* colonization in children is high with approximately 12% of asymptomatic *S. pyogenes* carriers among school children [17]. At the time of the suspected puerperal sepsis GAS outbreak the total number of iGAS infections in the Netherlands was increasing. Surveillance and subsequent typing of iGAS cases in 2022 by the NRLBM indicated that *emm*12 was the dominant *emm* type during the occurrence of the suspected *emm*12.0 outbreak [5]. The finding of a regional cluster suggests local transmission in the community may have contributed to the increase in puerperal sepsis cases that was noted in the LUMC. It is however not possible to rule out nosocomial transmission in one or two cases despite preventive measures.

In the first screening (round 1 and 2) only HCW with a link to more than one patient were included, later screening was broadened and all HCW employed at the maternity ward, obstetrics outpatient clinic, and fertility clinic were asked to participate in screening for GAS carriage. The participation rate of the untargeted screening was 66%, leaving about one third unscreened, which is in line with previous reports [18]. Screening for GAS carriage identified colonization in 4.1% of HCW, similar to 2% colonization in a meta-analysis [19]. All HCW were screened by nasal and pharyngeal swabs, a well-accepted screening method among HCW and the preferred method in earlier outbreaks. The probability of transmission of GAS is expected to be highest from skin, nose and throat. Potential anal or vaginal GAS carriage may have been missed, as earlier outbreaks studies have found such nosocomial sources [2, 20]. Current Dutch guidelines do however not indicate screening of vagina and anus, and after careful consideration the increase in sensitivity was anticipated to not outweigh the burden of vaginal screening on HCW and its impact on attendance rate [21, 22]. HCW were screened once, two months after occurrence of the last case, which may have resulted in failing to identify HCW carriers due to transient colonization [18]. We did identify two HCW carrying GAS belonging to the regional *emm*12.0 cluster. This indicates that the strain was present in the hospital during this cluster. But the cluster consisted of cases with and without a link to the hospital maternity ward. It remains unclear whether these HCW carriers were part of a transmission chain in the hospital or in the community. Overall, department-wide screening of all HCW did not contributed to the definite detection of a source, but did detect *emm*12.0 carriers. Treating these may have interrupted a part of the transmission chain. In January 2023, two more puerperal sepsis cases were identified, but both were of two different *emm* types, not being 12.0. On the other hand, no more regional, or even national, *emm*12.0 GAS cases were identified either, although the national incidence of puerperal sepsis continued to be elevated throughout 2023.

During this outbreak investigation, the obtained strains from initial case samples were typed using cgMLST at our local laboratory. Eventually, all strains were typed using both cgMLST at the LUMC and wgSNP analysis at the NRLBM. Both methods resulted in identification of the same isolates as part of the cluster, indicating that both typing methods can be reliably used for detecting clusters of *S. pyogenes*.

An important lesson learned from this outbreak is the added value of comparing iGAS cases across institutional boundaries and to similar infections in the community. This requires bacteriological molecular surveillance at a national level. When in the initial outbreak investigation, no obvious nosocomial source is detected, early collaboration to identify regional cases may give an indication of the potential presence of a community source or cluster. This can place an increased incidence of bacterial infections in the hospital in a regional context and may potentially prevent the use of impactful large-scale interventions or screening programs. In this particular outbreak a community cluster could have been a possible explanation for the increased incidence of puerperal sepsis at the maternity ward, though transmission within the hospital was another scenario. Therefore, the OMT nevertheless decided that an intervention was necessary. And indeed, no more related cases were diagnosed after this intervention.

## Conclusions

In conclusion, collaboration between hospital, PHS and the national reference laboratory visualized a cluster of puerperal sepsis cases and the presence of an outbreak strain in HCW carriers, but also revealed that community links might contribute more to the incidence of healthcare-associated infections than previously acknowledged. Here, WGS, using either cgMLST or wgSNP analysis, confirmed the presence of a regional *S. pyogenes* cluster, addressing a possible alternate source. Centralized collection of iGAS case information in combination with availability of molecular strain typing results is critical to place hospital clusters in the context of regional epidemiology. An increase in healthcare-associated infections may not necessarily imply an increase in healthcare-acquired infections.

## Electronic supplementary material

Below is the link to the electronic supplementary material.

Supplementary Material 1

## Data Availability

Sequence data have been deposited in the Sequence Read Archive (SRA) under project numbers PRJNA966900 and PRJNA967239.

## Abbreviations

cgMLST: Core-genome multilocus sequence typing
GAS: Group A Streptococcus
HCW: Healthcare worker
iGAS: Invasive group A Streptococcus
LUMC: Leiden University Medical Centre
NRLBM: Netherlands Reference Laboratory for Bacterial Meningitis
OMT: Outbreak management team
PHS: Public health services
S. pyogenes: Streptococcus pyogenes
WGS: Whole genome sequencing
wgSNP: Whole-genome single nucleotide polymorphism
