# Supplementary material for: Value of collaborative investigation by hospital infection control, public health services and a national reference laboratory during an increase in puerperal sepsis
Source: Antimicrob Resist Infect Control. 2025 May 28;14:60. doi: 10.1186/s13756-025-01564-z (PMC12121280; doi:10.1186/s13756-025-01564-z)

**ADDITIONAL FILE 1**

**Culture protocol**

Swabs were taken using eSwab medium (Copan Italia S.p.a., Brescia, Italy). The eSwab medium was plated on sheep blood agar with aerobic and anaerobic incubation, and Columbia CNA Agar with 5% 76 sheep blood, colistin and nalidixic acid with aerobic incubation. Agar plates were incubated for 48 hours at 35°C. Suspected *S. pyogenes* colonies with beta-haemolytic growth were confirmed with matrix-assisted laser desorption/ionisation time-of-flight mass spectrometry (MALDI-TOF MS) using the Microflex system (Bruker Nederland B.V., Leiderdorp, the Netherlands).

**Figure A1**

Whole Genome Single Nucleotide Polymorphisms (wgSNP) analysis of same *S. pyogenes* isolates and cases as in Figure 2. The same cluster of 13 cases is identified as with cgMLST (cluster 1).


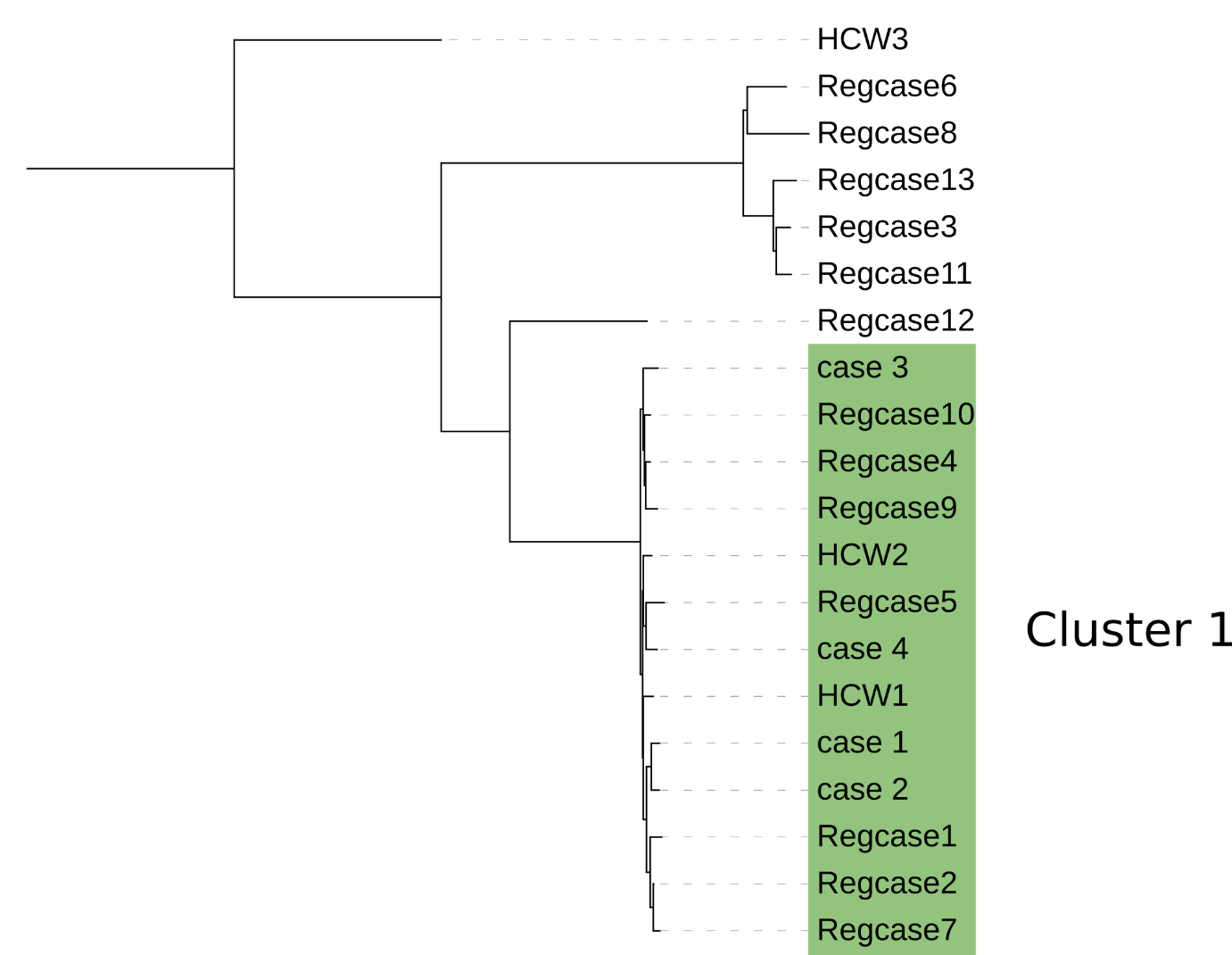

Supplement: Supplementary file 1 — Supplementary Material 1 [file 13756_2025_1564_MOESM1_ESM.docx]
